# Effects of marine sediment as agricultural substrate on soil microbial diversity: an amplicon sequencing study

**DOI:** 10.1186/s40793-023-00519-4

**Published:** 2023-08-07

**Authors:** Dámaris Núñez-Gómez, Pablo Melgarejo, Juan José Martínez-Nicolás, Francisca Hernández, Rafael Martínez-Font, Vicente Lidón, Pilar Legua

**Affiliations:** Centro de Investigación e Innovación Agroalimentaria y Agroambiental (CIAGRO-UMH), Miguel Hernandez University, Ctra. Beniel, km 3.2, Orihuela, Alicante, 03312 Spain

**Keywords:** Microbiome, 16S rRNA, Marine sediment, Agricultural substrate, Waste reuse, Functional inferences

## Abstract

**Background:**

The soil microbiota has a direct impact on plant development and other metabolic systems, such as the degradation of organic matter and the availability of microelements and metabolites. In the context of agricultural soils, microbial activity is crucial for maintaining soil health and productivity. Thus, the present study aimed to identify, characterize, and quantify the microbial communities of four types of substrates with varying proportions of marine port sediment used for cultivating lemons. By investigating microbial diversity and relative abundance, the work aimed to highlight the importance of soil microbial communities in agriculture when alternative culture media was used.

**Results:**

The composition and structure of the sampled microbial communities were assessed through the amplification and sequencing of the V3-V4 variable regions of the 16 S rRNA gene The results revealed a diverse microbial community composition in all substrate samples, with a total of 41 phyla, 113 classes, 266 orders, 405 families, 715 genera, and 1513 species identified. Among these, Proteobacteria, Bacteroidota, Planctomycetota, Patescibacteria, Chloroflexi, Actinobacteriota, Acidobacteriota, Verrucomicrobiota, and Gemmatimonadota accounted for over 90% of the bacterial reads, indicating their dominance in the substrates.

**Conclusions:**

The impact of the substrate origin on the diversity and relative abundace of the microbiota was confirmed. The higher content of beneficial bacterial communities for plant development identified in peat could explain why is considered an ideal agricultural substrate. Development of “beneficial for plants” bacterial communities in alternative agricultural substrates, regardless of the edaphic characteristics, opens the possibility of studying the forced and specific inoculation of these culture media aiming to be agriculturally ideals.

**Supplementary Information:**

The online version contains supplementary material available at 10.1186/s40793-023-00519-4.

## Introduction

Soils, considered one of the most dynamic and diverse ecosystems on the planet, can contain hundreds of thousands of bacterial species per gram of sample. This diversity, based on the presence and/or absence of microorganisms, can be related to the potential, current and/or historical use of the soil [1].

With the development and availability of new technologies based on genetic sequencing, it is possible to quickly characterize, identify and quantify millions of genomic sequences. Metagenomic studies, although complex, have been used successfully in different contexts and/or applications, such as medicine, animal production, pharmacological, polluted ecosystems, soils, and water, among others, which is undoubtedly increasing the accumulated knowledge about the bacterial microbiota, its interactions with the environment and other microorganisms [2–7].

Soil biota, which refers to the diverse community of microorganisms and macroorganisms that inhabit soil, plays a fundamental role in maintaining the key functions of soils. This is especially true for agricultural soils, where soil biota can directly impact plant development through processes such as organic matter degradation, nutrient cycling, and metabolite production. However, it’s important to note that soil biological fertility, which includes soil biota as well as other factors such as soil physical and chemical properties, is what ultimately determines the soil’s ability to support and sustain plant growth. Understanding the relationship between soil biota and soil biological fertility is crucial for promoting healthy soil and maximizing agricultural productivity [8, 9]. In addition, within the edaphic profile, the microbial activity in the first region of the soil, the rhizosphere, is essential for good vegetative and productive development. A great microbial diversity thrives in the rhizosphere in close association with plant roots where various abiotic and biotic interactions take place [10, 11].

From an agricultural point of view, it is necessary to know, identify and quantify the microbial characteristics of the soils and/or potential substrates for agricultural use in a specific and directed way, since these microbial communities are directly influenced by the physicochemical soil characteristics.

On the other hand, the higher price and depletion of peatland have highlighted the need to find new agricultural substrates suitable for crops [12]. In recent years, the European Union has been promoting, through projects support inside the LIFE and HORIZON 2020 frameworks, for example, the study, characterization and suitability of alternative substrates to peat from wastes. In this sense, and considering port dredging as a management and management activity necessary for the maintenance of the depth and therefore, the transit of large ships and the economic activity, the reuse and revalorization of the marine port sediment show promise. Multidisciplinary teams from Italy and Spain have been continuously studying the potential of phytoremediated marine sediment as an agricultural substrate. Their results have confirmed the correct vegetative and productive development of the plants when using the marine port sediment mixed with other substrates [13–16].

However, nothing is yet known about the influence and/or variation in the soil microbiota, this being a very important factor for the good vegetative development of plants. In this sense, the present work had as its main objective the identification, characterization and quantification of the microbial communities of four types of soils with different proportions of marine port sediment used to cultivate lemons. An exhaustive study on the functional inference of the identified bacterial communities was also performed.

## Materials and methods

### Soil samples

A marker-based approach using the 16 S ribosomal RNA subunit gene (rRNA16S) was used to study bacterial diversity. The soil samples were growth media for *Citrus limon* var. Verna. The 2-years-old lemon trees were cultivated in an experimental farm located in the Miguel Hernández University (38°04’05.8"N 0°58’56.5"W, Orihuela, Spain). The cultivation of lemon trees (*Citrus limon* L. Burm var ‘Verna’) began in May 2020 with planting and ended in January 2022 when the lemon fruits were harvested. A total of 90 lemon trees were used (30 lemon trees × 3 culture media), planted in pots with a maximum capacity of 40 L. The data regarding crop fertigation and lemon production can be consulted in Hernández et al. [17] and Martínez-Nicolás et al. [18], respectively. All the lemon trees were monitored and its crop was managed homogenous aiming to minimize the external impacts and variations. At the moment of soil collection, all the trees presented a good phytosanitary state.

For this study, three types of growing media were investigated with different marine port sediment content mixed with peat as shown in Table 1. A control soil sample (100% peat) was used aiming to identify the impact/modifications related with the marine port sediment content. This study did not consider a 100% port sediment sample because previous analyses demonstrated its infeasibility for use as an agricultural substrate because it caused a delay in the vegetative growth of the crop and a significant decrease in its production. For all the samples five replicates were used for microbial identification and statistical analysis. In total, 20 soil samples were analyzed in this study (4 soil samples x 5 replicates). The soil samples were collected from the cultivation pots after a two-year agronomic experiment with lemon trees. For each sample, 10 g of substrate were collected from the top 30 cm, as they correspond to the plant’s root development area. It is important to highlight that, in accordance with the objective of this study, the microbial characterization of the substrates was carried out after confirming the viability of the tested mixtures for the vegetative development of the trees, which was achieved after two years of cultivation. Furthermore, it is worth emphasizing that the purpose of this characterization was to study the microbiota of the substrates after their successful use as growing media, rather than their temporal evolution. Therefore, a single sampling was conducted to obtain this information.

The marine port sediment came from Italy (Livorno Port), and prior to its use, it was phytoremediated for 2 years [19], briefly: the phytoremediation treatment was conducted at a pilot scale using 12 not covered containers of about 1 m [3] each. The following plant treatments were tested: *Paspalum vaginatum, Phragmites australis, Spartium Junceum, P. vaginatum, Nerium oleander, P. vaginatum, Tamarix gallica, P. vaginatum*, and unplanted control. Sediment samples and leachate were periodically collected and analyzed for about two years. After that time, the plants were removed, and the sediment samples for each container were collected and characterized. The sediments were then subjected to a three-month period of landfarming aimed at further reducing organic contamination and improving their biological activities. Sediment samples were collected after 1.5 and 3 months of landfarming. At the end of the treatment, the marine port sediments presented good agronomical characteristics and were according to the Spanish and Italian legal limits. The same marine port sediment has already been used successfully, mixed with other substrates, for food, non-food and horticultural crops [13–15].

**Table 1:** Lemon cultivation soil samples used in this study with emphasis on the peat and marine port sediment content

	Grown media composition	
Sample ID	Marine port sediment content (%)	Peat content (%)
Control	0	100
S25	25	75
S50	50	50
S75	75	25

### rRNA sequencing

The composition and structure of the microbial communities were assessed by amplifying and sequencing the V3–V4 hypervariable regions of the 16 S rRNA gene using the 341 F/785R primer set. This primer set is one of the most extensively used sets for investigating bacterial diversity in various environments and was developed by Klindworth [20]. The Illumina Miseq sequencing 2 × 300 approach was used. The microbial samples were subjected to amplification using 25 PCR cycles, following the laboratory methodology recommended for moderate to high microbial biomass samples, such as soil [21]. A negative control of the DNA extraction was included as well as a positive Mock Community control (OMICS™ Microbial Community DNA Standard, Catalog Nos. D6305 and D6306) to ensure quality control.

### Bioinformatic processing and analysis

**Fig. 1:**
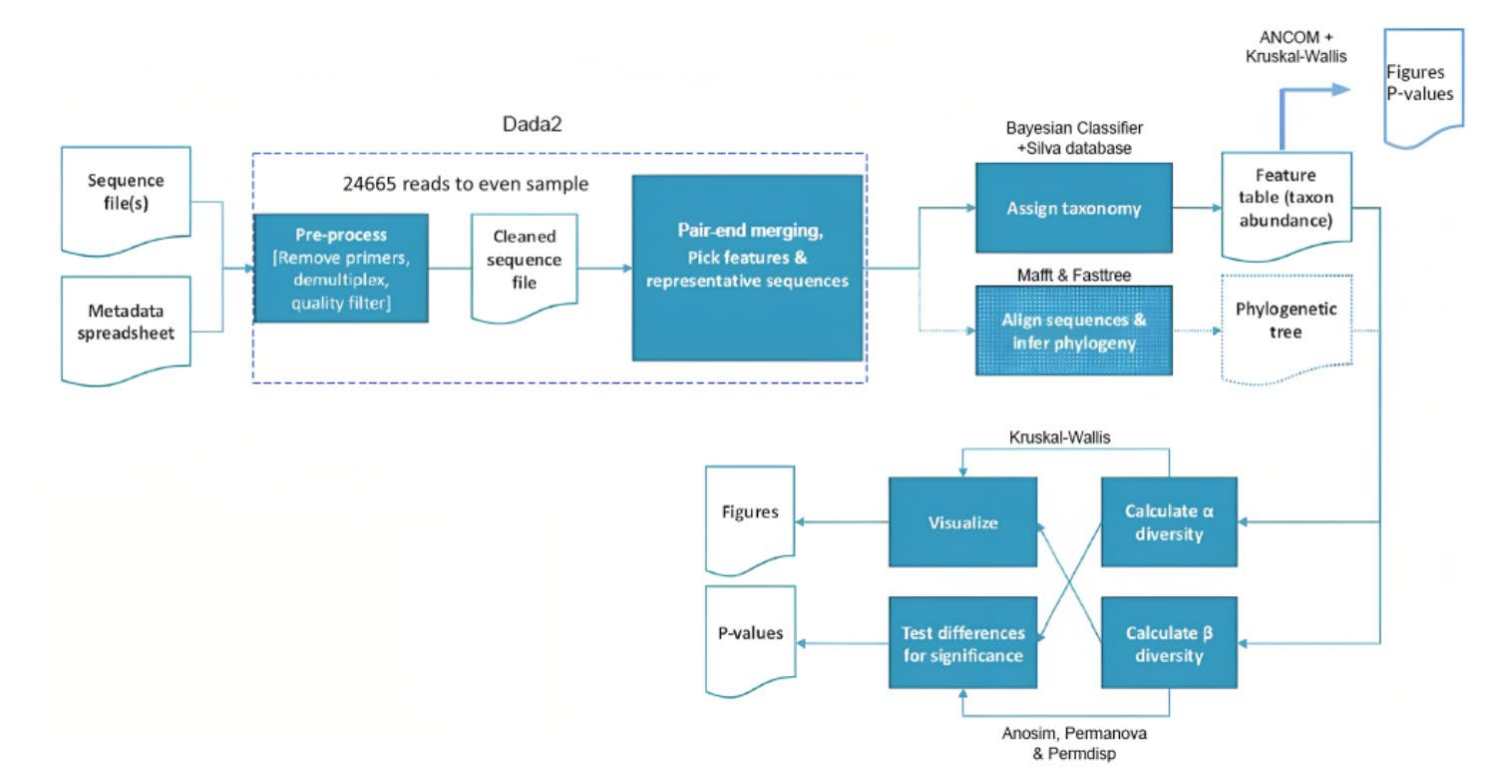
Sequence processing and analysis pipeline

Raw demultiplexed forward and reverse reads were processed with microbiome bioinformatics platform QIIME2 (https://library.qiime2.org) [22]. The open-source software package Dada2 (https://github.com/benjjneb/dada2) was used as QIIME 2 plugin for sequence quality control [23]. All the samples were subsampled up to 24,665 reads to even sample size and make quantitative comparisons. Phylotype data was used to calculate the following alpha diversity metrics: (i) Observed operational taxonomic unit (OTUs) (community richness); (ii) Evenness (or Pielou’s Evenness; a measure of community evenness); and (iii) Shannon’s diversity index (quantitative measure of community richness) [23]. Phylotype and phylogenetic data were used to calculate the following beta diversity metrics: (a) Unweighted Unifrac distance (phylogenetic qualitative measure); (b) Weighted Unifrac distance (phylogenetic quantitative measure); (c) Jaccard distance (qualitative measure); and d)Bray Curtis distance (quantitative measure) [24, 25]. Taxonomic assignment of phylotypes was performed using a Bayesian Classifier trained with Silva database version 138 (99% OTUs full-length sequences) [26]. Figure 1 shows the steps of the analysis.

### Statistical analysis with R software

As explained above, Alpha diversity comparisons were performed using Kruskal-Wallis non-parametric test and Beta diversity distance matrices were used to calculate principal coordinates analysis (PCoA) and to make ordination plots using R software package version 3.6.0 (R Foundation, Vienna, Austria) [27]. Significance threshold was set at 0.05. BiodiversityR version 2.11-1, PMCMR version 4.3, RVAideMemoire version 0.9-7 and vegan version 2.5-5 packages (https://www.r-project.org/) were used for the different statistical analysis carried out. The significance of groups present in community structure was tested using Permanova and ANOSIM tests [28]. In addition, a multivariate analogue of Levene’s test for homogeneity of variances, Permdisp test, was used to identify location vs. dispersion effects [28]. Significance threshold was set at 0.05. Differential relative abundance of taxa was tested using two methods: (i) the analysis of composition of microbiomes – ANCOM 29; and (ii) Kruskal Wallis non-parametric test [27]. After Kruskal Wallis, Conover’s test with FDR Benjamini-Hochberg correction was added for pairwise comparison [30].

### Functional inference of microbial communities

Potential functional profiles were predicted using PICRUSt2. Briefly, phylotypes were placed into a reference tree containing 20,000 full 16 S rRNA genes from prokaryotic genomes in the Integrated Microbial Genomes (IMG) database. Functional annotation of these genomes were based on the following biological databases: (i) The Kyoto Encyclopedia of Genes and Genomes (KEGG) orthologs (KO) (https://www.genome.jp/kegg/); (ii) Clusters of Orthologous Genes (COGs) (https://www.ncbi.nlm.nih.gov/research/cog); and (iii) Enzyme Commission numbers (EC numbers) [31, 32]. MetaCyc ontology (www.metacyc.org) predictions were used for inference of pathway abundances using MinPath [33, 34]. In order to infer Metacyc pathways, EC numbers were first regrouped to MetaCyc reactions. Pathway abundances were calculated as the harmonic mean of the key reaction abundances in each sample. To infer the abundance of each gene family per sample: the abundances of phylotypes are corrected by their 16 S rRNA gene copy number and then multiplied by their functional predictions.

## Results and discusion

### Quality control

As a consequence of the sequencing of the samples, the total number of sequences obtained was 2,008,938. To ensure appropriate quality, reads were trimmed in the position where phred quality dropped under Q20 (25th percentile), 288 nt for Forward and 226 nt for Reverse reads as shown in Figure S2a and Figure S2b (*Supplementary material*), respectively. In both cases, the plot at positions 288 (Forward reads) and 226 (Reverse reads), was generated using a random sampling of 10,000 out of 2,008,938 sequences without replacement. The minimum sequence length identified during subsampling was 301 bases. The number of reads obtained for the negative and Mock Community controls were as expected according to the report by the manufacturer (Fig. S1*Supplementary material*). The mock control was processed the same way as the samples.

The profile obtained was consistent to the theoretical expected, validating the processes of library preparation [35]. Bias are common and known between observed taxa in mock samples and theoretical expected mock composition. Negative control samples were used to detect environmental derived contaminants. In this sense, only three phylotypes were detected as contaminant amplicons and comprised less than two orders of magnitude difference between negative controls and samples, being these: phylotype *50f068f1f01312f274e181f1913ea912*, classified as genus *Saccharimonadales*, phylotype *765bcbd3b0c52b388ac6ab422de632ec* classified as family *Hyphomonadaceae* and phylotype *0a6c0a0801413c25e0f2de0602ddb63d* classified as family *Gemmatimonadaceae*. Results of differential abundance for these taxa were excluded.

### Diversity analysis

**Fig. 2:**
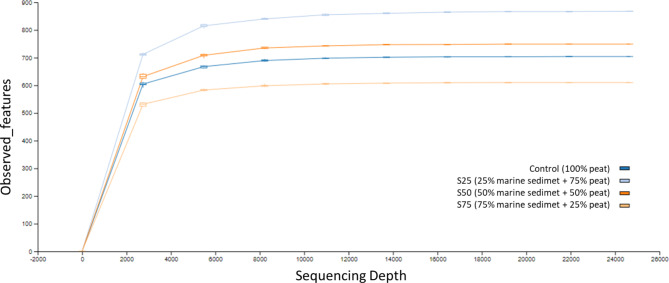
Rarefaction analysis plot of sequencing reads of the 16 S rRNA gene lemon cultivation soil samples

After quality control, 12,780 phylotypes were detected. Singletons and doubletons were also removed. The reading depth was confirmed adequate by the sequencing rarefaction curve, as shown in the Fig. 2. In this figure, it is observed that although not all the samples have the same number of reads, all the samples reach the maximum number of OTUs and sequencing depth does not result in an increase in the number of observed OTUs. Rarefaction curves confirm the adequacy of the reads [36].

#### Alpha diversity: within sample diversity

**Fig. 3:**
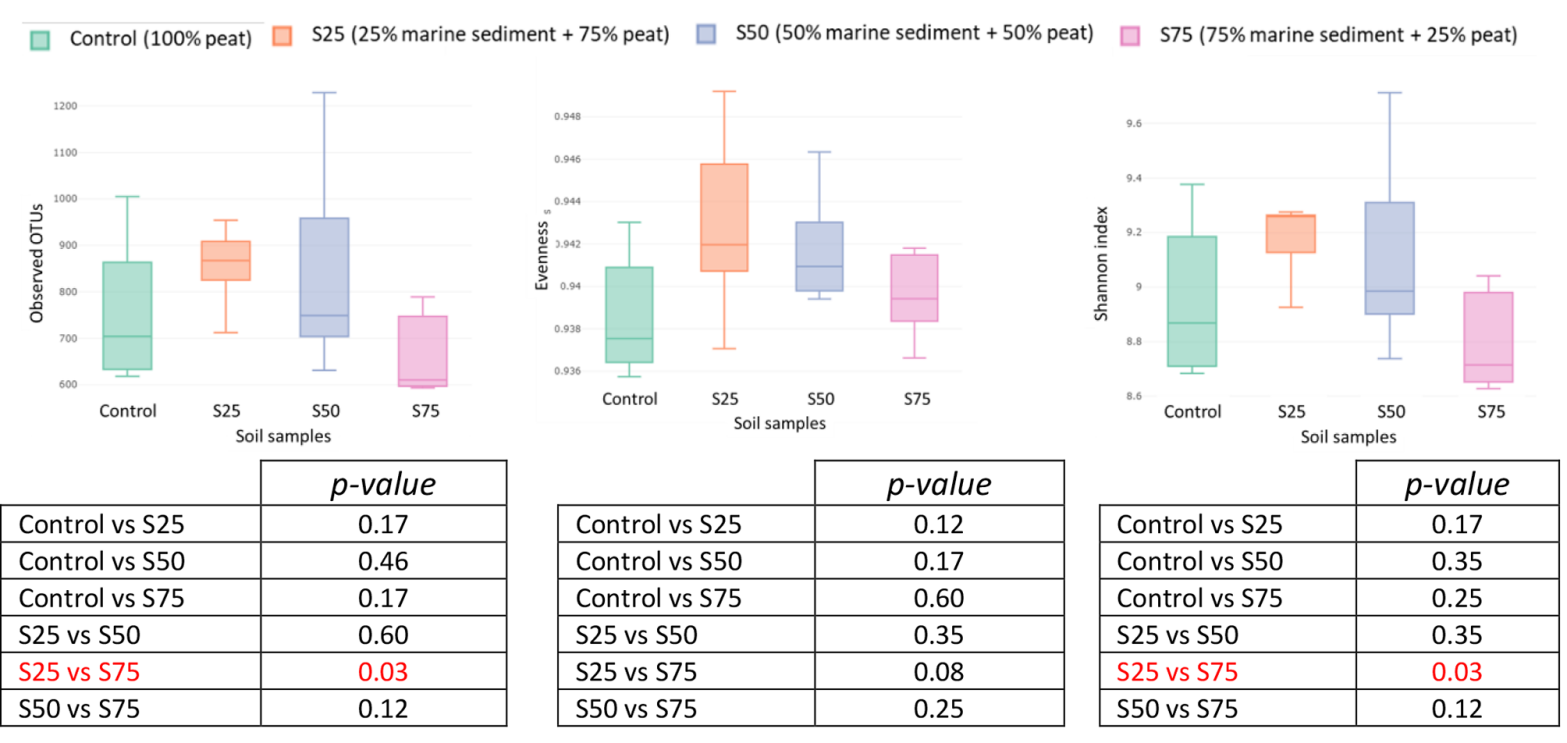
Boxplots of alpha diversity metrics (Observed OTUs, Evenness, and Shannon index) used to study the metagenomic of lemon production soil samples. The results highlighted in red indicate significant differences between experimental groups according to the Kruskal-Wallis and post-hoc tests (two-by-two comparisons controlling for significance)

For the diversity within a sample measure, alpha diversity, the richness or observed OTUs (defined as the number of different species/phylotypes are present in a community), the evenness (used to quantify how equal the community is numerically) and the Shannon index (combines richness and diversity) were calculated (Table 2; Fig. 3). The results indicated significant differences between the S75 and S25 soil samples in terms of richness (observed OTUs). The S75 soil sample showed lower richness (664.60 OTUs) compared to the S25 soil sample (857.80 OTUs) as observed in Fig. 3. In all the soil samples, the communities were numerically similar, with evenness values ≥ 0.939. However, the homogeneity of the abundance of the communities in the samples, evaluated by the Shannon index, indicated that the soil samples S25 (9.182) and S50 (9.113) presented a greater presence of species with a well-balanced abundance than S75 and Control, where the Shannon index values were not higher than 8.954 (Table 2). Significant differences (p < 0.05) between samples, evaluated by the Kruskal-Wallis test, were only identified between samples S25 and S75 for the observed OTUS and Shannon index. The evenness results did not show significant differences between the soil samples (Fig. 3). It is worth highlighting that the mere fact of maintaining and observing similar bacterial communities across all samples is a positive outcome, as it demonstrates the viability of using small quantities of port sediment mixed with peat.

**Table 2:** Alpha diversity parameters for 16 S rRNA of lemon cultivation soil samples composed of different peat and marine sediments proportions

Soil Sample^*^	Observed OTUs	Evenness	Shannon Index
S25	857.80 (89.25)	0.943 (0.004)	9.182 (0.15)
S50	841.00 (232.7)	0.942 (0.003)	9.113 (0.37)
S75	664.60 (90.41)	0.940 (0.002)	8.801 (0.19)
Control	756.20 (159.12)	0.939 (0.003)	8.954 (0.29)

^*^S25; S50, and S75 indicate 25%, 50%, and 75% marine sediment content, respectively. Control = 100% peat. The results represent the mean value (n = 5) and standard deviation

#### Beta diversity: between samples diversity

**Fig. 4:**
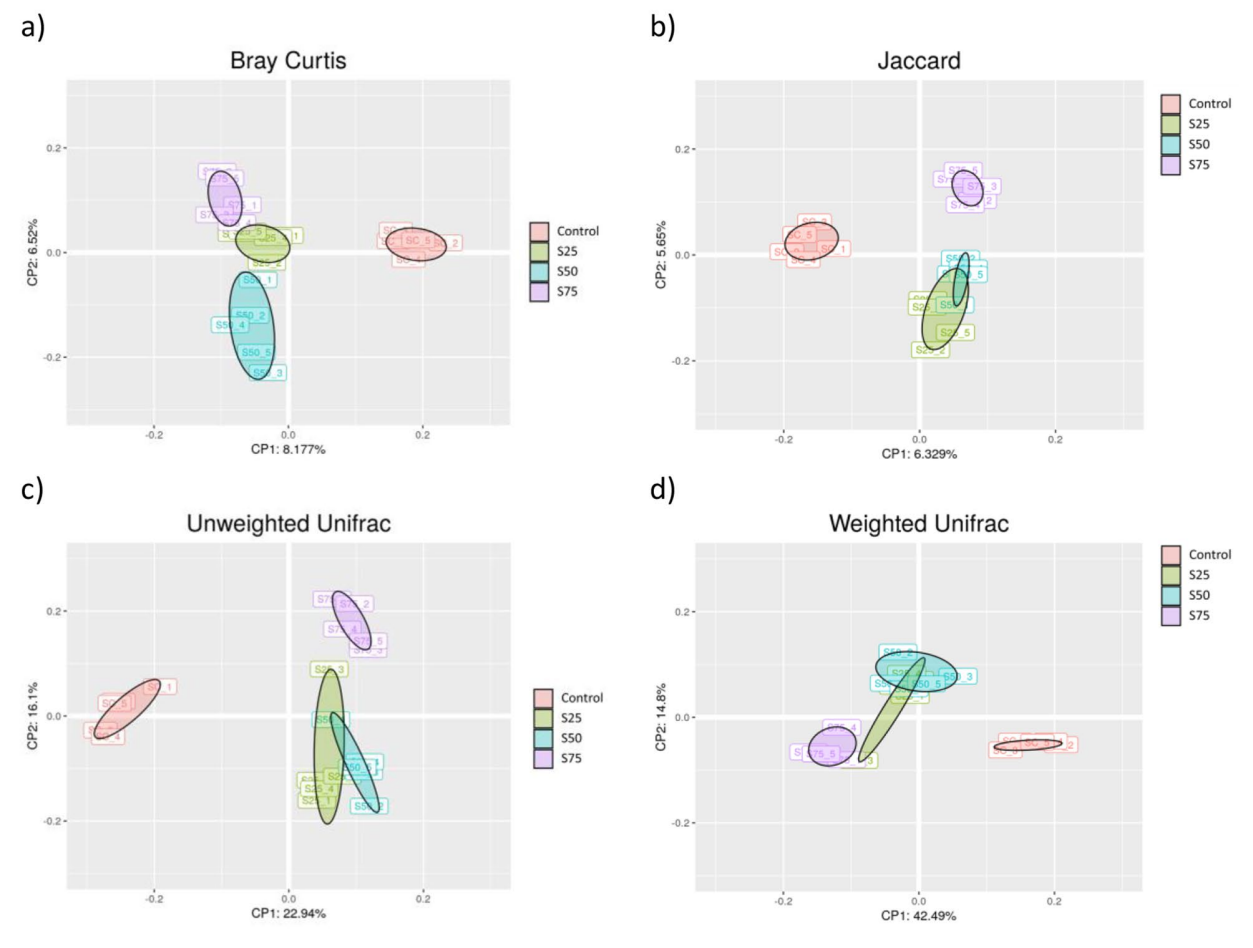
Principal coordinate analysis (PCoA) of microbial composition using Bray-Curtis (a), Jacccard (b); Unweighted Unifrac (c); and Weighted Unifrac (d)

Beta diversity measures differences in the composition of the microbiome between samples [36]. To measure the similarity between two microbial compositions, various ecological distances or differences can be used. The most commonly used quantitative measures include Bray-Curtis and Jaccard. Additionally, phylogenetic quantitative measures, such as unweighted UniFrac and weighted UniFrac distances, can be used to account for evolutionary relatedness between microbial communities [37–39]. The visualization of microbial communities’ structure results was done through PCoA plots (Fig. 4). Each dot represents one sample replicate, and distances between dots represent the ecological distances between samples. Each replicate of samples was assigned a different colour [40]. The results of the applied metrics showed a good separation between the soil samples, being higher in the case of the Bray-Curtis method (Fig. 4a). In the other cases, the S25 and S50 groups clustered at a similar level, which would indicate that the bacterial communities were similar among them (Fig. 4b, c and d). On the other hand, the grouping distance between the control sample (100% peat) and the soil samples with different marine port sediment content would clearly indicate the differentiation between the bacterial communities identified in the control from the rest of the samples. This finding would support the idea that soil type has a significant impact on bacterial communities.

### Taxonomic profile. Relative abundance

For the lemon cultivation soil samples, with different proportions of marine port sediment, a total of 41 phyla, 113 classes, 266 orders, 405 families, 715 genera and 1513 species were identified in all the soil samples.

The dominant phyla bacterial in all soil samples were *Proteobacteria, Bacteriodota, Planctomycetota, Patescibacteria, Chloroflexi, Actinobacteriota, Acidobacteriota, Verrucomicrobiota* and *Gemmatimonadota*, accounting for more than 90% of the identified bacterial reads (Fig. 6). Previous studies on the characterization of marine port sediments also identified the *Proteobacteria* phylum as predominant, and associated the *Gemmatimonadot*a phylum with plants and rhizosphere ecosystem because this phylum can do anoxygenic photosynthesis [41, 42]. In addition, the *Bacteroidota* phylum is reported in all ecosystems but mainly in soils, and is defined as a digester of complex carbohydrates-based biomass, contributing to a wide range of biogeochemical processes, including soil formation, soil fertility, and carbon storage [43].

The phyla *Myxococcota, Crenarchaeota, Cyanobacterias* and *Bdellovibrinota* were minor phyla (abundance > 1%) in soil samples with marine port sediment (S25 > S50 > S75), while in the control sample (100% peat) presented values > 1% but < 5%. These differences would confirm, as expected, the higher predisposition of peat than marine port sediment as an agricultural substrate since these phyla have already been reported as essential in the microbial profile of agricultural land [44].

*Deinococcota* phylum was not identified in Control samples only was detected in soil samples with marine port sediment, with a relative abundance proportional to marine port sediment content (S75 > S50 > S25), indicating that can be related to the marine port sediment and not with the peat, once that phylum is positively related to the apparent density of the soil, they can exist and grow in extreme environments (humidity, temperature, etc.) [45]. On the contrary, *Iainarchaeota* phylum was only identified in the control samples, while *Spirochaetota* was only identified in the control and S50 samples. Youssef et al. [46] defined *lainarchaeota* as free-living organisms capable of fermenting a limited range of substrates, such as ribose, polyhydroxybutyrate, and some amino acids while Magot et al. [47] related the proliferation of *Spirochaetota* with environments contaminated by petroleum and metal ions. In the S75 samples were the only group in which *Desulfobacterota* and *Latescibacterota* were not detected. RCP2-54 was only detected in S25 soil samples.

**Fig. 5:**
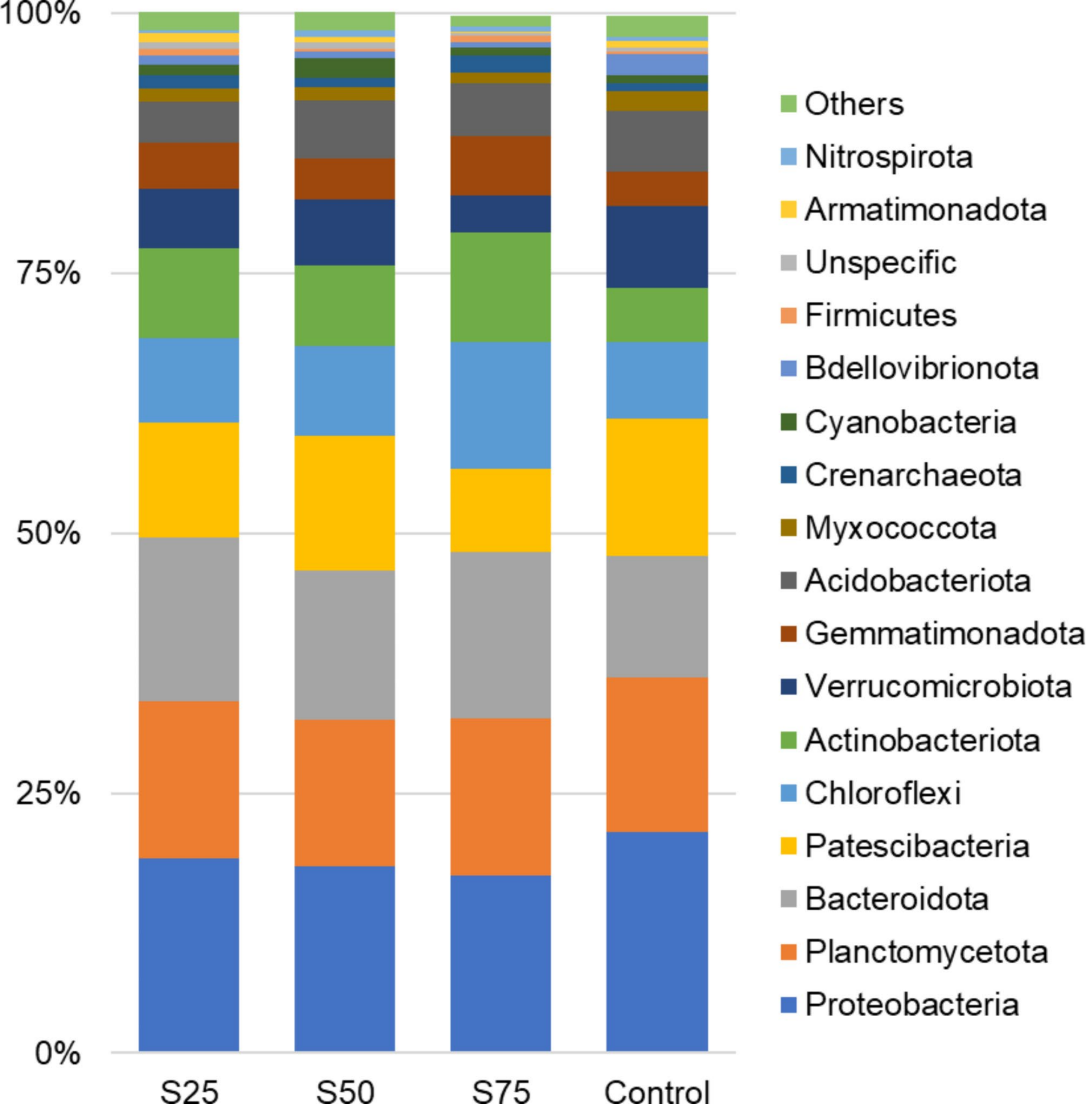
Bar plots of the Relative Abundance for Phylum level for the lemon cultivation soil samples

It was observed that the abundance of *Proteobacteria* (Control: 21.23%; S25: 18.70%; S50: 17.93%; S75: 17.02%), Patescibacteria (Control: 13.26%; S25: 12.99%; S50: 10.92%; S75: 7.96%), *Verrucomicrobiota* (Control: 7.97%; S25: 6.37%; S50: 5.75%; S75: 3.61%),*Bdellovivrionota* (Control: 2.05%; S25: 0.85%; S50: 0.53%; S75:0.44%), and *Myxococcota* (Control: 1.89%; S25: 1.39%; S50: 1.31%; S75: 1.00%) phyla, was inversely proportional to the content in marine port sediment, while the abundance of *Chloroflexi* (Control: 7.27%; S25: 8.20%; S50: 8.66%; S75: 12.17%), *Actinobacteriota* (Control: 5.21%; S25: 7.71%; S50: 8.58%; S75: 10.56%), and *Gemmatinmonadota* (Control: 3.25%; S25: 3.87%; S50: 4.48%; S75: 5.69%) phyla increased as the content of marine port sediment in the culture substrate increased (Fig. 5). The identification of bacterial phyla with a marine origin in the culture substrate could confirm the evidence of their direct relationship with port sediment, which is responsible for their introduction into the substrate. This finding underscores the remarkable adaptability, resistance, and persistence capacity of these bacteria in their new ecological niche. Despite a previous two-year phytoremediation effort, the persistence in the substrates suggests that these bacterial phyla possess robust adaptive mechanisms to withstand environmental stressors. In this sense, Coolen et al. [48] characterized the marine sediments of the eastern Mediterranean and determined that more than 70% of the bacteria, when the sediments were rich in organic matter, belonged to the phylum *Choroflexi* or non-sulfur green bacteria. On the other hand, the phylum *Acidobacteria* could be attributed as a contribution of the peat, since it is not usually found in marine habitats [42], but also this phylum is related to beneficial relationships with plants, regulation of biogeochemical cycles, decomposition of biopolymers, exopolysaccharide secretion, and plant growth promotion [44, 49].

**Fig. 6:**
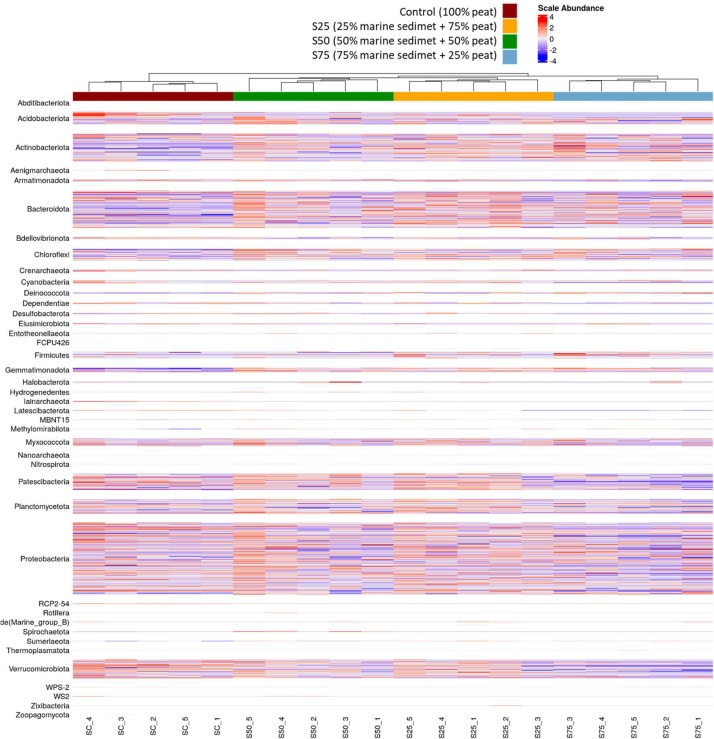
Heatmap plot and dendrogram of bacterial genus identified in the five replicates of each studied soil sample (Control as SC, S25, S50 and S75)

The most abundant bacterial genus, that were possible to classify from all the reads, correspond to *Actinobacteriota* genus *Nocardioides* (S75 > S50 < S75 < Control), *Bacteriodota* genus *Salinimicrobium* (S75 > S50 < S75 < Control); *Bdellovibrionota* genus *Bdellovibrio* (Control > S25 > S50 > S75); *Chloroflexi* genus JG30-KF-CM45 (S75 > S50 < S75 < Control) and AKYG1722 (S75 > S50 < S75 < Control); *Cyanobacteria* genus *Chloroplast* (S50 > S25 > S75 > Control); *Gemmatimonadota* genus S0134_terrestrial_group (S75 > S50 < S75 < Control); *Patescibacteria* genus *Saccharimonadales* (S50 > S75 > S50 > Control); *Planctomycetota* genus WD2101_soil_group (S75 > Control > S50 > S25); *Proteobacteria* genus *Sphingomonas* (S75 > S50 < S75 < Control) and *Pseudomonas* (Contol = S50 > S25 > S75); *Verrucomicrobiota* genus *Pedosphaeraceae* (S25 > S50 > S75 > Control) (Fig. 6). The results are consistent with the scientific literature since the main genera identified in S75 samples are related to coastal sediments, saline environments and/or coral ecosystems [50–52], while the most abundant in the control samples (100% peat), such as *Pseudomonas* and *Bdellovibrio*, stand out for their potential as biofertilizers due to its metabolic pathways produce and secrete plant growth regulators such as auxins, gibberellins and cytokinins, improving processes such as seed germination, mineral nutrition, root development, use of water, among others [53, 54].

**Fig. 7:**
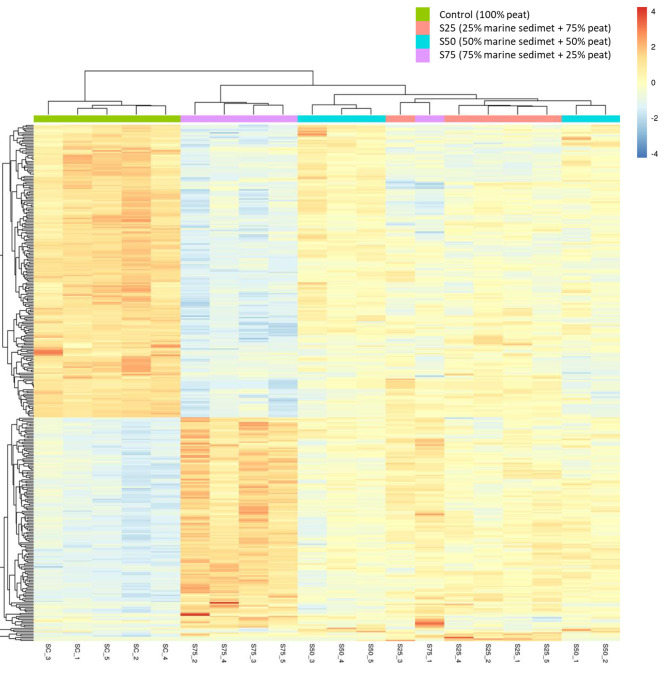
Heatmap plot resulted from the analysis of differential relative abundances of COG functions between samples levels (n = 5) performed using Kruskal Wallis test

On the other hand, the most abundant specific genus for each of the soil samples, that is, genera that were only detected in one sample, were: Acidobacteriota genus Solibacter, Bacteriodota genus Terrimonas and Kapabacteriales, Gemmatimonadota genus YC-ZSS-LKJ147, Patescibacteria genus Candidatus_Peregrinibacteria and Candidatus_Adlerbacteria, Planctomycetota genus CPla-3_termite_group, Proteobacteria genus Reyranella, Acidibacter, Rhodanobacter and Devosia; Verrucomicrobiota genus Opitutus and Ellin517 in the control samples which agrees with reported for peat-based agricultural substrates in different environmental conditions and geographical locations 55,56; Bacteriodota genus Pedobacter, Patescibacteria genus Candidatus_Campbellbacteria in S25 soil samples both related with organic matter decomposition 45; and in S75 soil samples, Bacteriodota genus Antarcticibacterium and Planctomycetota genus SH-PL14 reported for marine sediments, with high tolerance to salt [57, 58], and Proteobacterias genus Lysobacter most referenced for its positive synergies to plant growth due to the activity of its metabolites/enzymes against bacteria, fungi, oomycetes and some nematodes [59, 60]. Inside the Archaea phylum, Crenarchaeota genus Nitrososphaeraceae was the most abundant in all the soil samples, with a higher abundance in S75 than Control samples which would confirm the predominance of ammonia-oxidizing environments in S75 samples [61] (Fig. 7). The results indicate a clear differentiation between the soil samples although all the samples have been subjected to the same conditions (climate and lemon crop management), with the only difference related to the marine port sediment content.

### Functional profiles

Although the rarefaction curves showed that the quality reads did not include 100% of the OTUs, the asymptotic trend of the rarefaction curves confirmed the representativeness of the sampling and therefore the characterization of the bacterial communities in the samples.

#### Analysis based on clusters of Orthologous genes (COGs)

Clusters of Orthologous Groups (COG) is defined as a database based on the identification of the protein systems of the complete genomes of bacteria, algae, and eukaryotes and each COG group is composed of orthologous sequences so that the function of the sequence can be inferred [62].

Analyzing the results with the COG database, 4291 protein sequences with correspondence to a COG number were identified in the bacterial genome of the control samples (100% peat), while for the samples containing marine port sediment the number of correlated sequences was slightly higher, 4373, 4326 and 4312 for S75, S50 and S25, respectively.

By inferring the function of the identified proteins and classifying them, the results showed that of all the protein functions, those related to Cell wall/membrane/envelope biogenesis (M) functions was the functional group most represented, followed by Transcription (K) > Lipid transport and metabolism (I) > Signal transduction mechanism (T) > Carbohydrate transport and metabolism (G) > General function prediction only + Coenzyme transport and metabolism (HR) indicating good metabolomic activity in the samples [63]. However, significant differences were observed between the soil samples according to the Kruskal-Wallis test (Fig. 7). In this sense, in the control samples, the greatest relationships were attributed to the Cell wall/membrane/envelope biogenesis and Signal transduction mechanism functions, while in the S75 samples were mostly related to Signal transduction devices + Transcription and Lipid transport and metabolism which would indicate soil samples with different metabolic functions according to the environmental conditions, where the proliferation and nutrition functions were predominant for control and S75 respectively [64, 65]. For S25 the functions related to Transcription were the most abundant. According to Zhou et al. [66] transcription function as predominant in a microbial community could indicate continuous microorganism responses to internal and/or external environmental changes aiming to optimize its metabolism and guarantee its survival (Fig. 7).

#### Analysis based on the Kyoto Encyclopedia of genes and genomes (KEGG)

**Fig. 8:**
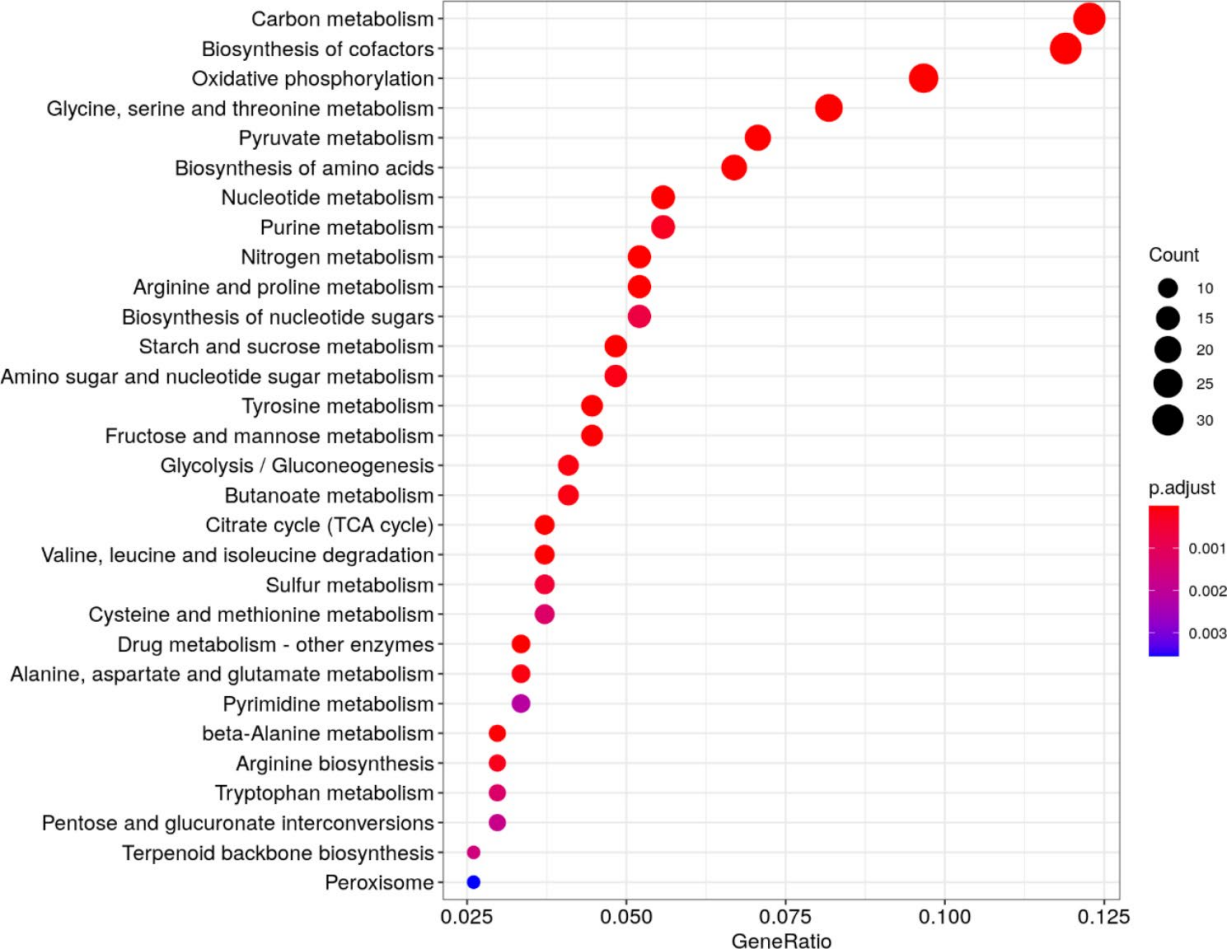
Variable Importance Projection (VIP) scores of more significant metabolic pathways, according to Fisher’s exact test, identified for the lemon cultivation soil samples. Over represented KEGG pathways. Bar length comprises the count of KO functions observed in each pathway

To identify the metabolic pathways involved, taxonomic profiling was performed on soil samples and the results were compared to the KEGG database. Of the 3514 metabolic pathways identified in the soil samples studied, only 939 exhibited significant differences in their relative abundances among the different treatments, as determined by the Kruskal-Wallis test. Enrichment analysis was performed on the significant pathways using Fisher’s test to interpret the results better. Based on this analysis, 61 pathways were defined as more enriched, and among those, 30 pathways were identified as dominant, with a relative abundance greater than 1.00%, and correlated mainly with carbon metabolism, biosynthesis of cofactors, oxidative phosphorylation, glycine, serine and threonine metabolism, pyruvate metabolism and biosynthesis of amino acids (Fig. 8). The results are consistent with those reported in the literature for different types of soils, such as agricultural soils, bioremediated and/or recovered soils, and marine/coastal sediments [63, 67, 68].

## Conclusion

The metagenomic analysis conducted in this study revealed that substrate origin has a significant impact on the diversity and relative abundance of microbiota present in agricultural substrate. Specifically, differences were observed in the diversity and abundance of microbiota present in soils derived from different substrates. Furthermore, the study found that peat, which is commonly considered an ideal agricultural substrate, contains a higher proportion of beneficial bacterial communities compared to substrates containing port sediments. These beneficial bacterial communities play a crucial role in supporting plant development and growth. This study represents a pioneering effort in developing and characterizing an alternative substrate to peat following its successful implementation in a citrus crop for a duration of over two years. The research is firmly rooted in previously published studies and the wealth of knowledge acquired, encompassing both the sediment and its agronomic potential. This work is the first of its kind to focus on verifying and comprehending the metagenomic variability exhibited by the substrate itself, with a comparative analysis against the reference substrate, namely peat. The study also demonstrated the possibility of maintaining “beneficial for plants” bacterial communities in substrates with marine port sediment, regardless of the edaphic characteristics. This finding opens up the possibility of further investigation into forced and specific inoculation of these substrates to promote healthy plant growth. In conclusion, the findings of this study provide valuable insights into the impact of substrate origin on the microbiota in agricultural soil, and highlight the importance of careful selection of substrates to support healthy plant growth. These findings may have important implications for the agricultural industry, and further research is warranted to explore the potential of forced and specific inoculation of substrates to promote beneficial bacterial communities in agricultural substrates.

### Electronic supplementary material

Below is the link to the electronic supplementary material.

Supplementary Material 1

## Data Availability

Not applicable.
