# Supplementary material for: Effects of marine sediment as agricultural substrate on soil microbial diversity: an amplicon sequencing study
Source: Environ Microbiome. 2023 Aug 7;18:69. doi: 10.1186/s40793-023-00519-4 (PMC10408225; doi:10.1186/s40793-023-00519-4)
Supplement: Supplementary file 1 — Supplementary Material 1 [file 40793_2023_519_MOESM1_ESM.docx]

Supplementary Material

Fig. S1: Theoretical composition of the mock community in terms of 16SrRNA gene abundance as reported by the manufacturer (a) and historical observed relative abundance of reads in mock samples assigned up to Genus level using Silva database ^1^ (b).


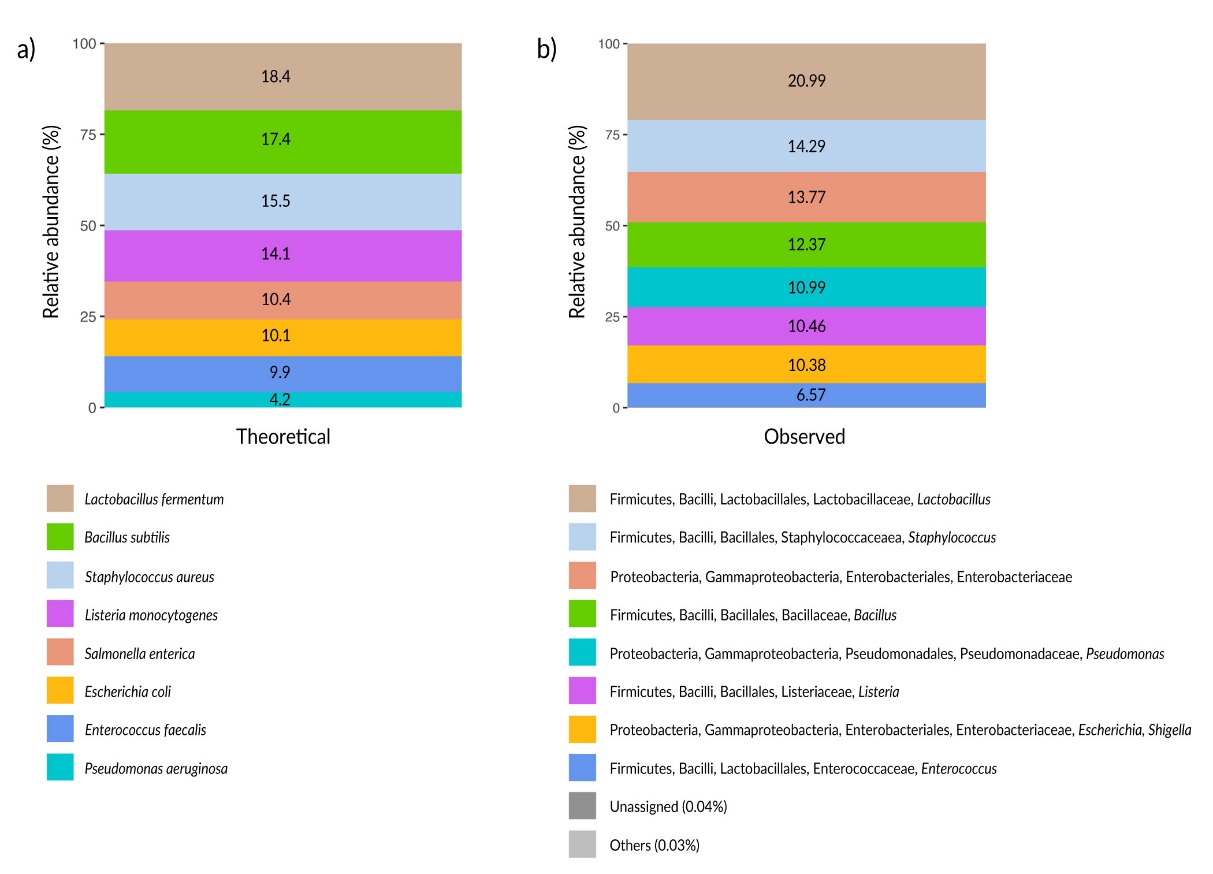


Fig. S2 Fig. 2. Sequence quality for Forward (a) and Reverse (b) Reads.

| a) |
| --- |
| 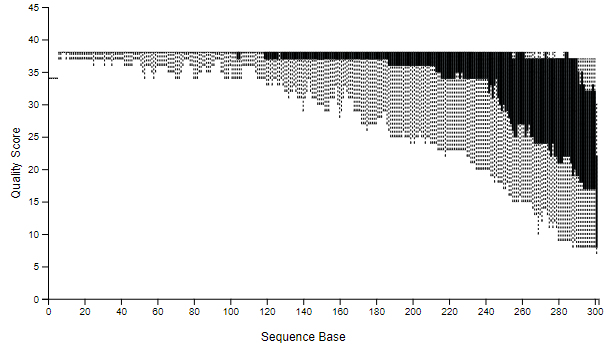 |
| b) |
| 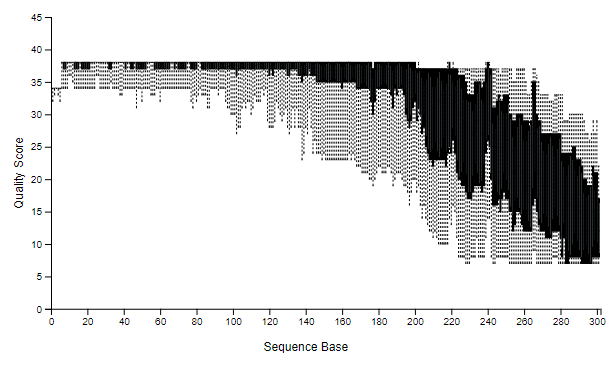 |

**REFERENCES**

1. Wang, Q., Garrity, G. M., Tiedje, J. M. & Cole, J. R. Naïve Bayesian Classifier for Rapid Assignment of rRNA Sequences into the New Bacterial Taxonomy. *Appl Environ Microbiol* **73**, 5261–5267 (2007).
